# Role of Innate Interferon Responses at the Ocular Surface in Herpes Simplex Virus-1-Induced Herpetic Stromal Keratitis

**DOI:** 10.3390/pathogens12030437

**Published:** 2023-03-10

**Authors:** Jiayi Ren, Ferrin Antony, Barry T. Rouse, Amol Suryawanshi

**Affiliations:** 1 Department of Pathobiology, College of Veterinary Medicine, Auburn University, 240B Greene Hall, Auburn, AL 36849, USA; 2 College of Veterinary Medicine, University of Tennessee, Knoxville, TN 37996, USA

**Keywords:** HSV-1, HSK, HSE, IFN-α, IFN-β, IFN-λ, type I IFNs, type III IFNs, ISGs, TLRs

## Abstract

Herpes simplex virus type 1 (HSV-1) is a highly successful pathogen that primarily infects epithelial cells of the orofacial mucosa. After initial lytic replication, HSV-1 enters sensory neurons and undergoes lifelong latency in the trigeminal ganglion (TG). Reactivation from latency occurs throughout the host’s life and is more common in people with a compromised immune system. HSV-1 causes various diseases depending on the site of lytic HSV-1 replication. These include herpes labialis, herpetic stromal keratitis (HSK), meningitis, and herpes simplex encephalitis (HSE). HSK is an immunopathological condition and is usually the consequence of HSV-1 reactivation, anterograde transport to the corneal surface, lytic replication in the epithelial cells, and activation of the host’s innate and adaptive immune responses in the cornea. HSV-1 is recognized by cell surface, endosomal, and cytoplasmic pattern recognition receptors (PRRs) and activates innate immune responses that include interferons (IFNs), chemokine and cytokine production, as well as the recruitment of inflammatory cells to the site of replication. In the cornea, HSV-1 replication promotes type I (IFN-α/β) and type III (IFN-λ) IFN production. This review summarizes our current understanding of HSV-1 recognition by PRRs and innate IFN-mediated antiviral immunity during HSV-1 infection of the cornea. We also discuss the immunopathogenesis of HSK, current HSK therapeutics and challenges, proposed experimental approaches, and benefits of promoting local IFN-λ responses.

## 1. Introduction

HSV-1 is a double-stranded DNA virus that infects and replicates in epithelial cells of the orofacial mucosa. The virus is a highly prevalent human pathogen, and a recent estimate suggests that globally 3.7 billion people are seropositive for HSV-1 [1,2]. The primary infection usually occurs early in life through close contact of infectious viral particles with orolabial mucosal epithelial cells [1]. These days, HSV-1 is also a common cause of genital infections [2,3]. HSV-1 is a neurotrophic virus and establishes latency in the peripheral nerve ganglia [4]. After initial lytic replication in mucosal epithelial cells in the facial region, HSV-1 enters sensory neurons and travels to the TG, establishing lifelong latency [4,5,6]. Once infected, unlike love, it lasts forever [4,6,7]. The clinical outcome of HSV-1 infection largely depends on the site of recurrent HSV-1 replication and the host’s immune status [6,8]. Although recurrent HSV-1 infection of the orofacial region causes mild herpes labialis, repeated recurrences in the cornea can result in a chronic inflammatory reaction, referred to as HSK, that impairs vision and can cause blindness [1,9]. Clinically, HSK is characterized by severe ocular pain, foreign body sensation, redness, photophobia, corneal opacity, watery discharge, corneal ulceration, scar formation, and gradual vision loss [10,11]. If left untreated, HSK can deteriorate into necrotizing keratitis with a significantly increased risk of corneal melting and perforation [10,12,13]. Corneal HSV-1 infection and associated HSK pathology is a leading cause of infectious blindness in the United States [13,14,15]. Globally, 1.5 million cases of ocular HSV-1 infection are reported annually, with 40,000 new cases of vision impairment and blindness [1,14,15]. 

Past studies in murine models of primary and recurrent HSV-1 infection have defined HSK as immunopathological conditions mainly driven by initial viral replication (epithelial keratitis with dendritic lesions) in the corneal epithelium followed by uncontrolled activation of innate and adaptive immune responses [16,17,18]. The recurrent HSV-1 infection in the cornea leads to progressive corneal scarring, neovascularization, and damage to the sensory neurons that can result in neurotrophic keratopathy [17,19,20]. The lytic HSV-1 replication in epithelial cells activates host defense mechanisms primarily driven by the production of IFNs, chemokines, and cytokines [17,18,21,22]. These initial antiviral responses promote the infiltration and activation of innate immune cells (neutrophils, macrophages, natural killer (NK) cells, dendritic cells (DCs), and subsequently the induction of CD4^+^ and CD8^+^ T-cell-mediated adaptive immunity and antibody responses [18,23,24,25,26,27,28,29]. The innate type I and type III IFNs play a pivotal role in clearing the infection [21,22,30,31]. Type I IFNs are produced by all virus-infected nucleated cells, whereas type III IFNs are predominantly the product of virus-infected mucosal epithelial cells [21,22,32,33,34]. Although the induction of early innate IFN responses is essential for HSV-1 clearance, the subsequent uncontrolled activation of both innate and adaptive immune responses can cause irreversible damage to the cornea and lead to vision impairment [18,21,32,35]. Innate IFNs not only participate in initial HSV-1 clearance but also function to regulate the induction of adaptive immune responses via several mechanisms still being investigated [8,21,22,32,36]. The optimal induction of these innate IFN responses commonly succeeds in restricting HSV-1 replication and can be lethal in patients with genetic deficits of innate immunity, particularly those relating to the production of or response to IFNs [37,38,39]. Indeed, HSV-1 expresses numerous viral proteins and has evolved multiple successful strategies to counteract innate IFN responses to evade host immunity [40,41,42]. In this review, we summarize the current understanding of the HSV-1 replication cycle and recognition of HSV-1 pathogen-associated molecule patterns (PAMPs) by different host PRRs. Furthermore, we discuss the induction and role of innate type I and type III IFN responses as well as downstream IFN-stimulated genes (ISGs) in the control of HSV-1 replication. We also discuss HSV-1-mediated immune evasion strategies that regulate PRR-mediated IFN responses after corneal HSV-1 infection. Finally, we discuss the pathogenesis of ocular HSV-1 infection and progression to HSK, current HSK treatment approaches and challenges, and an alternate IFN-λ therapeutic strategy that could simultaneously promote both antiviral and anti-inflammatory responses during recurrent HSK.

## 2. HSV-1 Entry, Replication, Assembly, and Egress

**Figure 1:**
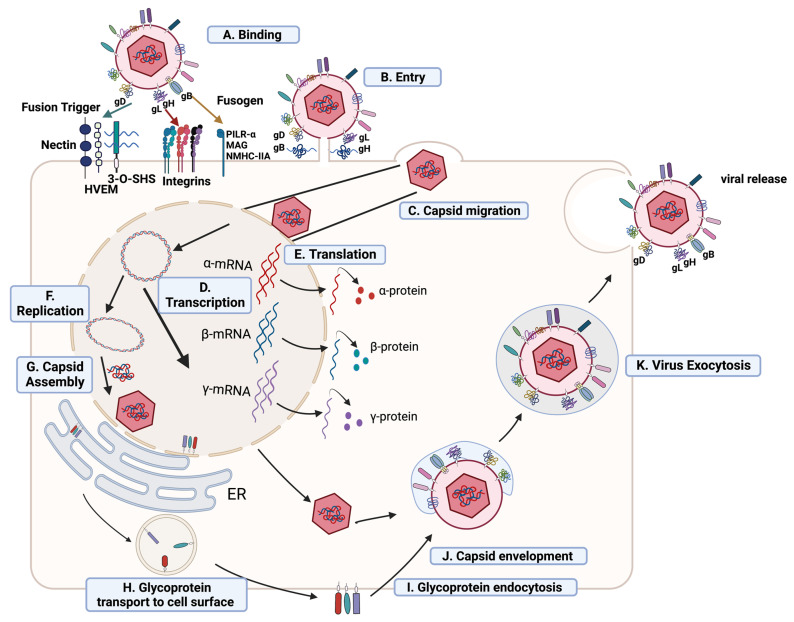
HSV-1 infection and lytic replication. (**A**) Binding. The interaction of HSV-1 gD with host cell receptors recruits a fusion complex comprised of gB, gH, and gL to combine viral and cellular membranes. (**B**) Entry. HSV-1 capsid and tegument proteins are released into the host cell cytoplasm. (**C**) Capsid migration. HSV-1 tegument proteins promote capsid transport to the nucleus, where viral DNA enters the nucleus through the nuclear pores. (**D**) Transcription. HSV-1 genes (α, β, and γ) are sequentially transcribed in the nucleus. (**E**) Translation. HSV-1 mRNAs are transported from the nucleus to the cytoplasm for translation and protein synthesis. (**F**) Replication. HSV-1 genome replicates as a rolling-circle. (**G**) Capsid assembly. Capsid proteins are transported into the nucleus for capsid assembly along with the HSV-1 genome. (**H**) Glycoprotein transport to the cell surface. Glycoproteins are exported from the trans-Golgi network to the cell surface. (**I**) Glycoprotein endocytosis. The glycoproteins from the cell surface are endocytosed to synthesize viral envelope in the cytoplasm. (**J**) Capsid envelopment. Glycoproteins fuse with capsid in the cytoplasm. (**K**) Viral exocytosis. Coating of capsid with glycoproteins leads to the release of virions into the extracellular space. (Created with BioRender.com).

The HSV-1 virion comprises three parts: the virion core (double-stranded DNA genome surrounded with an icosahedral capsid), a protein-containing layer called the tegument, and an outer lipid layer envelope [43]. The HSV-1 genome is 152–155 kbp in length, which includes unique long and short segments (UL and US) flanked by inverted repeats [43,44]. The tegument layer comprises a minimum of 20 viral and cellular proteins that are essential for viral replication [44]. The outer virion envelope is derived from the cell membrane and is embedded with 11 viral-encoded glycoproteins (gB-gM) [43,45]. The initial HSV-1 attachment to the host cell is mediated through the interaction between the cell heparin sulfate proteoglycans and viral gB and gC [45]. Subsequently, the viral gD interacts with host cell receptors such as nectin-1, nectin-2, herpes virus entry mediator (HVEM), and 3-O-sulfated-heparan sulfate (SHS) [46]. The interaction of gD with host cell entry receptors recruits a fusion complex comprised of gB, gH, and gL to the host cell membrane [47,48,49]. Furthermore, gB interacts with host cell receptors, such as paired immunoglobulin like-type 2 receptor-α (PILR-α), Myelin-associated glycoprotein (MAG), and non-muscle myosin heavy chain IIA (NMHC-IIA) [45,49,50,51] (Figure 1).

The ocular HSV-1 tropism is determined by the expression of entry receptors such as HVEM and nectin-1 [52,53]. Accordingly, a deficiency of HVEM and nectin-1 in mice makes them resistant to corneal HSV-1 infection [53]. Once HSV-1 is inside the host cell, tegument proteins interact with host cell motor proteins (dynein, dynactin, and kinesin) to promote capsid transport to the nucleus [54]. Viral proteins, such as pUL36 and pUL37, play a central role in transporting capsids to the nucleus [55,56,57,58]. Once the capsid reaches the nucleus, viral DNA enters the nucleus through a nuclear pore, followed by viral mRNA transcription by host RNA polymerase II and viral proteins [59,60]. 

The viral mRNA transcription, DNA replication, and DNA encapsidation occur in the nucleus through a controlled cascade of processes. During lytic replication, HSV-1 genes are expressed temporally in three distinct classes critical for viral replication, assembly, packaging, and egress of viral particles [61,62]. These are immediate–early (IE), early (E), and late (L) genes, also referred as α, β, and γ genes [61,62]. The HSV-1 lytic cycle lasts for around 18 h. The IE protein synthesis occurs within 2–4 h post-infection (pi), E proteins within 5–7 h pi, and the L proteins within 12–16 h pi [63]. Initially, six IE genes, namely, infected cell protein ICP0, ICP4, ICP22, ICP27, ICP47, and US1.5, are transcribed without viral protein synthesis [64,65]. These IE genes drive the transcription of E genes, whose protein products are critical for viral DNA replication. After productive DNA replication, the transcription and translation of L genes are initiated [66,67,68]. The protein products of L genes, which are structural proteins, play a role in DNA replication and are critical for progeny viral particle assembly [68]. L genes are divided into two classes, γ1 and γ2, that encode the critical proteins, virion protein 16 (VP16), VP22, gB, and gC [68,69]. 

After viral DNA replication and viral protein translation, host cell proteins such as importin α-1 play a crucial role in viral protein importation into the nucleus, followed by capsid assembly and egress [70]. The initial assembly of the viral genome and nucleocapsid occurs in the nucleus, followed by the final assembly in the cytoplasm [71]. The nuclear inner membrane serves as the primary envelope during egress, but this is lost during fusion with the outer nuclear membrane, and capsids are then released into the cytoplasm [72,73,74]. The exit of viral capsids from the nucleus is initiated by the L gene protein products pUL31 and pUL34 which interact with viral and cellular proteins [75]. The capsid acquires its tegument and envelope in the cytoplasm by fusing with the trans-Golgi network vesicles and endosomes [76]. The pUL36 and pUL37 interact with kinesin, and this interaction plays a crucial role in viral transport from the nucleus toward the periphery [55,76,77]. The mature HSV-1 virions egress from infected cells via exocytosis (Figure 1) [78]. The host enzymes heparanase and cathepsin L are transported to the infected cell surface and play a role in the detachment of mature viral particles by cleaving heparan sulfate [79]. The HSV-1 virus DNA replication, assembly, and egress processes have been extensively reviewed [43,59,71,72,76,79,80,81]. 

During lytic HSV-1 infection, the replicating virus in the orofacial region epithelial cells promotes cell death in mucosa and skin with subsequent release of progeny viral particles. This uncontrolled viral replication and associated epithelial cell death results in fluid-filled blisters and other inflammatory signs [1,13,16]. Following rapid, productive HSV-1 replication in epithelial cells, HSV-1 enters the nerve endings of peripheral sensory neurons and undergoes retrograde transport to the TG [82,83,84,85]. HSV-1 establishes lifelong latency in the TG, where the HSV-1 genome is maintained in a non-replicating chromatin-associated state with minimal viral gene transcription and translation [82,83]. The HSV-1 latency and reactivation mechanisms have been extensively studied, yet still are incompletely understood [83,84,85]. Primary ocular HSV-1 infections are rare but can occur through direct inoculation or HSV-1 replication in orofacial epithelial cells, followed by transport to TG via the ophthalmic nerve [86]. However, the most common form of ocular HSV-1 infection is a sequel to reactivation of HSV-1 from the latently infected TG [86]. The following sections summarize how HSV-1-associated PAMPs during binding, entry, and replication stages are recognized by different cell surface, endosomal and cytosolic PRRs and how this serves to promote innate IFNs and downstream ISG production to control HSV-1 replication and spread. 

## 3. HSV-1 Recognition by the Host Immune System

The first line of defense against invading pathogens is anatomic and chemical barriers that prevent the pathogen’s access to susceptible epithelial cells. Moreover, epithelial cells of the cornea and mucosal surfaces are joined together by tight junctions, creating an obstacle for pathogens to pass [87,88,89,90]. Furthermore, these epithelial cells secrete antimicrobial proteins (AMPs) and mucins that inhibit the binding of pathogens to the cell surface [91,92,93,94]. One such AMP, LL-37, is produced by the corneal epithelium, and LL-37 can blunt infection by disrupting the viral envelope, preventing HSV-1 from binding to and infecting host epithelial cells [95,96]. Once HSV-1 binds and enters, lytic replication occurs rapidly, causing cell death and the release of progeny viral particles that can infect nearby uninfected cells [9,18]. This lytic replication and epithelial cell death breaches the physical barrier, and HSV-1 gains access to underlying tissue, such as the corneal stroma. However, innate immune cells, such as neutrophils, macrophages, and DCs, patrol the subepithelial tissue compartment and can sense the active infection by PRRs [9,18,97,98]. The PAMPs and damage-associated molecular patterns (DAMPs) generated by the infection act as ligands for PRRs, and the interaction initiates a cascade of signaling events that promote the production of IFNs and inflammatory cytokines [99,100,101,102,103]. This recognition of PAMPs and DAMPs results in further recruitment of innate immune cells that may act to initiate pathogen clearance [99,101,102,104]^,^. 

Based on the cellular expression site, PRRs are broadly divided into membrane-associated PRRs and cytosolic PRRs [99]. PRRs consist of five distinct families of receptors based on protein domain homology [101]. These are Toll-like receptors (TLRs), C-type lectin receptors (CLRs), AIM2-like receptors (ALRs), retinoic acid-inducible gene I (RIG-I)-like receptors (RLRs), and nucleotide-binding domain, nucleotide-binding oligomerization domain (NOD)-like receptors (NLRs) [101]. TLRs and CLRs are either expressed on the cell surface or associated with intracellular endosomal vesicles [102]. These membrane-associated PRRs detect extracellular pathogens on either cell surfaces or phagocytosed pathogens in the phagolysosomal compartment [103]. NLRs, RLRs, and ALRs are cytosolic PRRs and recognize intracellular pathogens and their nucleic acids in the cytoplasmic compartment [101,102,103]. This compartmentalization of PRRs is critical for the efficient detection of pathogens at various stages of the infection cycle, such as extracellular (cell-membrane-associated TLRs: TLR-1, TLR-2, TLR-4, TLR-5, and TLR-11/12), internalization (endosomal: TLR-3, TLR-7, TLR-8, TLR-9, and TLR-13), and replication (cytosolic PRRs detecting nucleic acids) [102,105]. Microbial PAMPs recognized by PRRs include lipoproteins, lipopolysaccharides (LPSs), and nucleic acids (RNA and DNA) [101,102]. PRR signaling in various cells promotes the activation of the transcription factors (nuclear factor kappa-light-chain-enhancer of activated B cells (NF-κB), activator protein 1 (AP-1), and interferon regulatory factors (IRFs)) [106,107]. This results in the increased transcription and production of cytokines, chemokines, AMPs and antiviral IFNs [105,106]. Moreover, PRR signaling promotes cellular processes such as phagocytosis, antigen (Ag) processing, and Ag presentation on major histocompatibility complex (MHC) molecules and their cell surface expression. Additionally, inflammasome pathway activation occurs as does increased cell surface expression of B7 molecules (CD80, CD86) on Ag-presenting cells (APCs), migration of APCs to local draining lymph nodes (DLNs), autophagy, and eventually death of infected cells [105,108,109]. 

**Figure 2:**
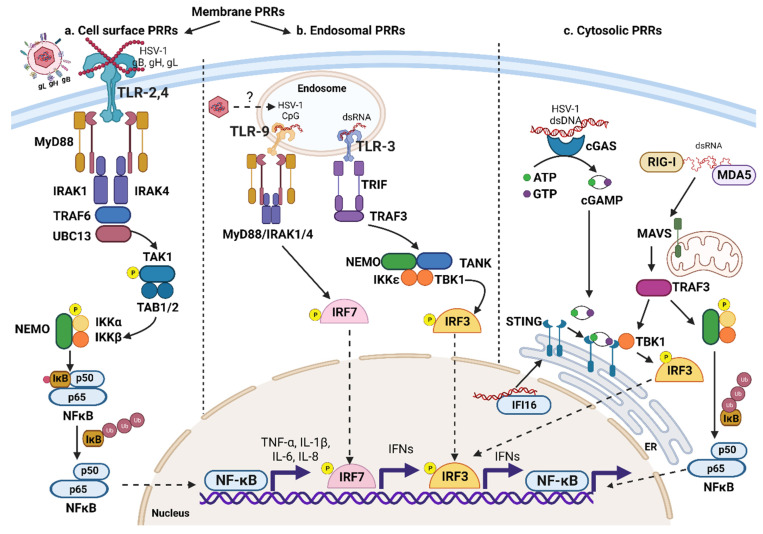
HSV-1 recognition by PRRs. HSV-1 is recognized by membrane-associated and cytosolic PRRs. HSV-1 bind and activate cell surface and endosomal TLRs to initiate a cascade of downstream signaling events resulting in the induction of inflammatory and antiviral responses. (**a**) TLR-2 via MyD88/IRAK1/4 signaling activates the transcription factor NF-κB, which in turn initiates inflammatory cytokine transcription. (**b**) HSV-1 CpG DNA activates TLR-9, whereas dsRNA activates TLR-3. Downstream activation of IRF7 or IRF3 leads to the production of IFNs. However, molecular mechanisms for HSV-1 recognition by TLR-9 and TLR-3 are unclear. Further, (**c**) cytosolic PRRs such as cGAS/STING and RIG-I/MDA-5 recognize HSV-1 CpG DNA and dsRNA, respectively, for the induction of IFNs and inflammatory cytokines. (Created with BioRender.com).

A common theme of TLR signaling is cell surface dimerization upon ligand binding, followed by the recruitment in the cytoplasmic compartment of adaptor protein to the TIR (Toll-IL-1 Receptor) domain of TLRs (Figure 2) [102,106,110]. All TLRs, except TLR3, recruit myeloid differentiation primary response 88 (MyD88) as an adaptor protein (Figure 2) [111,112]. TLR3, however, uses TIR-domain-containing adaptor-inducing IFN-β (TRIF) as an adaptor protein to initiate a downstream signaling cascade [111,112]. MyD88 has a TIR domain in the carboxy terminus and a death domain in the amino terminus [106,110]. Upon ligand binding and dimerization of TLRs, the TIR domain of MyD88 interacts with the TIR domain located in the cytoplasmic tail of TLRs [106,110]. The death domain of MyD88 binds to the death domain of IRAK (interleukin (IL)-1-receptor-associated kinase) 4 and IRAK1, activating these serine–threonine protein kinases [106,110]. Subsequently, the IRAK1-4 complex forms a signaling scaffold that recruits tumor necrosis factor receptor-associated kinase 6 (TRAF6), an E3 ubiquitin ligase [110]. In the next step, the TRAF6, in cooperation with ubiquitin-conjugating enzyme 13 (UBC13, E2 ubiquitin ligase), generates a polyubiquitin scaffold that further recruits a signaling complex consisting of TAK1 (transforming growth factor-β-activated kinase 1, a serine-threonine kinase) and polyubiquitin-binding proteins (TAK-1-binding proteins: TAB1 and TAB2) [106,110]. The activated TAK1 then binds to the IKK (IκB kinase) complex consisting of IKKα, IKKβ, and IKKγ (NF-κB essential modifier or NEMO), followed by TAK1-mediated phosphorylation and activation of IKKβ [106,110]. The NF-κB transcription factor consists of two subunits, p65 and p50, maintained in an inactive state through binding to the IκB (inhibitor κB) [113]. The activated IKKβ phosphorylates IκB and targets it for ubiquitin-mediated degradation, which results in the activation of NF-κB followed by its translocation to the nucleus, where it promotes the transcription of various target genes [114]. The endosomal TLR-3, TLR-7, TLR-8, and TLR-9 sense pathogen-associated nucleic acids and activate IRF, resulting in the increased expression of type I IFNs [100,105,110]. TLR-3 recognizes double-stranded RNAs (dsRNA) in the endosomal compartment, and this recruits the TRIF/TRAF3 complex activating IRF3 [99,102,105,110]. Both TLR-7 and TLR-8 recognize single-stranded RNA (ssRNA), whereas TLR-9 recognizes unmethylated CpG dinucleotides in the endosomes. TLR-7 and TLR-9 signaling recruits the MyD88/IRAK1/4 complex and activates IRF7 [102,105,110,115,116]. IRFs that are kept inactive in the cytoplasm are phosphorylated by TLR-mediated signaling. This results in activation and translocation of IRFs to the nucleus and the subsequent transcription of type I IFNs (Figure 2) [117,118].

In the cornea, HSV-1 primarily replicates in epithelial cells, where different PRRs recognize various HSV-1-associated PAMPs [119,120]. Corneal epithelial cells express numerous TLRs that include TLR-1, 2, 3, 4, 5, 7, 8, and 9 [121,122,123]. When HSV-1 infects, its glycoproteins gB, gH, and gL can interact with TLR-2 and activate NF-κB signaling [124]. Accordingly, as Sarangi et al. showed, mice lacking TLR-2, MyD88, and TLR-9 were resistant to HSK progression [125]. However, viral titers in the TG and cornea early after HSV-1 infection (day 3–4 pi) were comparable to controls in all strains of knockout mice [125]. Interestingly, TLR-2^-/-^ and MyD88^-/-^ mice showed increased periocular disease suggesting a critical role of TLR-2 in restricting HSV-1 replication in corneal epithelial cells, perhaps through the recruitment of innate immune cells and production of inflammatory cytokines [125]. In contrast, TLR-4-deficient mice showed early onset and increased HSK lesion severity indicating that TLR-4 activation may be necessary for producing anti-inflammatory cytokines and the induction of regulatory T cells that minimize lesions [125]. In addition, to increased lesion severity, MyD88^-/-^ mice showed increased susceptibility to encephalitis after corneal HSV-1 infection [125]. Furthermore, measurement of HSV-1 levels showed increased spread and persistence in the brain, indicating a critical role for TLR-induced innate and adaptive immune responses in limiting HSV-1 central nervous system (CNS) infection [125]. Based on the current paradigm, methylated and CpG motif-containing HSV-1 DNA can act as a ligand for TLR-9. However, the molecular mechanisms of TLR9-mediated sensing of HSV-1 DNA in the endosomal compartment is incompletely understood [98]. TLR-9 activation promotes type I IFN production as shown by Zheng et al. [126]. HSV-1 DNA can also promote neovascularization in the cornea. Thus, the TLR9 activity of HSV-1 DNA is proinflammatory and angiogenic, both relevant steps in the pathogenesis of HSK [127]. Furthermore, Wuest et al. showed a crucial role of TLR-9 and type I IFN signaling in the induction of the chemokine (C-X-C motif) ligand 9 (CXCL9) and CXCL10 production needed to recruit inflammatory cells after corneal HSV-1 infection [128]. 

Apart from TLR-9, HSV-1 DNA is also recognized by the cyclic GMP-AMP synthase (cGAS), a cytosolic dsDNA sensor, and interferon-inducible protein 16 (IFI16), a nuclear dsDNA sensor (Figure 2) [129,130,131,132,133]. The allosteric dsDNA binding to cGAS activates its catalytic activity, leading to the synthesis of 2′3′ cyclic GMP-AMP (cGAMP), a second messenger molecule and potent agonist of the stimulator of IFN genes (STING) [132,133]. Upon ligand binding, STING, which is an endoplasmic reticulum (ER) membrane protein, translocates to the Golgi and recruits TANK-binding kinase 1 (TBK1) to initiate downstream signaling [129,132,133]. The STING–TBK1 complex then recruits and phosphorylates IRF3/7 to induce type I IFNs and inflammatory cytokine production [130,134,135]. Royer et al. showed that STING^-/-^ mice are more susceptible to HSV-1 infection and that was evident by day 5 pi in the cornea [136]. Although STING deficiency led to a minimal decrease in the expression of ISGs in HSV-1-infected corneas, a significant reduction in the expression of antiviral effectors was observed in corneal epithelial cells [136]. This may be explained by a recent study showing that HSV-1 upregulates tripartite motif-containing protein 21 (TRIM21) expression in the corneal epithelial cells which can act to suppress the STING–IRF3 axis and the production of type I IFNs [137]. This study also showed that HSV-1-induced TRIM21 expression in epithelial cells promotes HSV-1 replication and IL-6 and TNF-α production resulting in the exacerbation of epithelial keratitis [137]. In addition, IFI16 is critical for IFN-β production after viral DNA recognition in the nucleus, and this recruits STING to activate IRF3 and NF-κB after HSV-1 infection [131,138]. Recently, it was shown that HSV-1 genome binding to IFI16 promotes antiviral responses and inhibits viral gene expression [139,140]. These studies demonstrated that IFI16 binds to the HSV-1 genome in a sequence-independent manner with enrichment at the DNA polymerase gene (UL30; catalytic subunit), acting to promote epigenetic silencing of the HSV-1 genome and consequently suppressing HSV-1 protein expression and replication [139]. Furthermore, Conrady et al. showed that IFI16 is crucial for the initial control of HSV-1 in the corneal epithelium, whereas TLR recognition of HSV-1 was expendable [141]. DEAD-box helicase 41 (DDX41) is an enzyme that plays a critical role in dsDNA homeostasis and regulates type I IFN responses by modulating activation of the cGAS–STING pathway [142]. In addition, the DNA-dependent activator of the interferon regulatory factor (DAI) recognizes the cytosolic B-form DNA, and this, in turn, activates IRF3 and NF-κB to promote type I IFN production [143]. However, during HSV-1 infection, DAI suppresses HSV-1 replication independent of DNA sensing through regulation of ICP0, which in turn promotes IFI16 degradation [144]. 

There are several other PRRs that affect the outcome of HSV-1 infection. These include RLRs that detect cytosolic 5′-triphosphate or 5’-diphosphate-containing short dsRNA as well as long dsRNA by RIG-I and melanoma differentiation-associated gene 5 (MDA-5) receptors [145,146,147]. Upon stimulation, RIG-I and MDA-5 recruit the adaptor protein mitochondrial antiviral-signaling protein (MAVS), which activates the TBK1 and IKK complex to activate IRF3 and NF-κB transcription factors. This results in the production of IFNs and inflammatory cytokines (Figure 2) [145,146,147,148]. Although the role of RLRs in recognizing RNA viruses is well recognized, their role in recognizing DNA viruses is less clear [147,148]. An earlier study suggested that MDA-5 and MAVS are the primary inducers of IFNs, acting independently of RIG-I signaling in primary human macrophages after HSV-1 infection [149]. Moreover, the knockdown of MDA-5 resulted in decreased IFN-β and IFN-λ production after HSV-1 infection in human macrophages [149]. However, emerging evidence suggests that HSV-1 infection induces the relocalization of the host 5S ribosomal pseudogene (RNA5SP141) from the nucleus to the cytoplasm and this results in host protein synthesis shutoff [150]. This process serves to deplete the RNA-binding proteins permitting RNA5SP141 to bind to RIG-I [150]. Furthermore, cytosolic AT-rich viral DNA is converted to 5’-ppp RNA species via RNA polymerase III (RNA Pol-III), which serves to activate RIG-I signaling [151]. Therefore, both host and viral RNA species can activate RNA Pol-III/RIG-I signaling [146]. Moreover, Liu et al. showed that the RIG-I signaling is critical for the STING pathway activation after HSV-1 infection [152].

In summary, these studies show that HSV-1 infection of the host cell is detected by multiple cell surface, endosomal, and cytosolic PRRs binding to viral proteins and nucleic acids at various stages of the HSV-1 replication cycle. Furthermore, numerous PRRs simultaneously or sequentially stimulate robust type I and type III IFNs, inhibit HSV-1 replication, and promote inflammatory cytokine and chemokine production to activate innate and adaptive immune responses. Although the role of TLRs and the STING pathway is well characterized during HSK, further studies are warranted to understand the role of other PRRs in initiating innate antiviral responses after corneal HSV-1 infection. In the following sections, we will briefly summarize the current understanding of type I and type III IFN-mediated signaling, the induction of ISGs, innate IFN responses in the cornea after HSV-1 infection, and how these innate IFN responses modulate HSV-1 replication and HSK progression. 

## 4. Type I and III IFN Responses 

**Figure 3:**
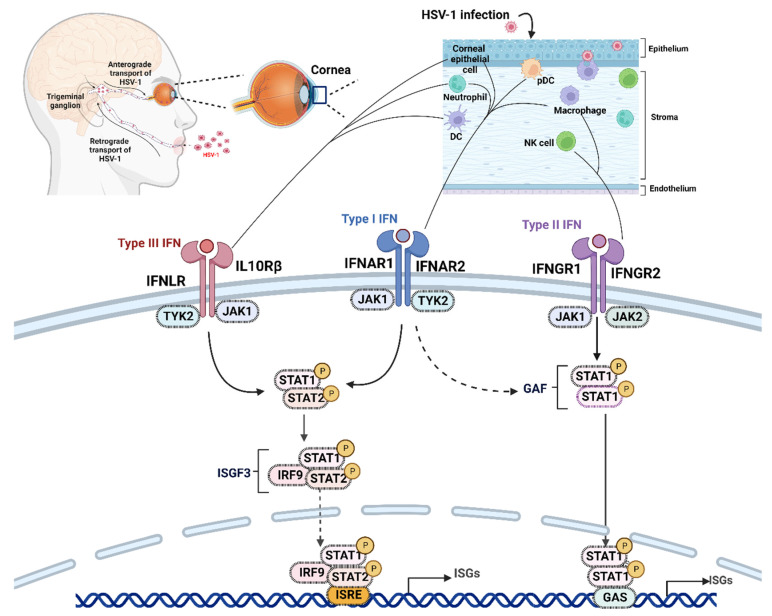
IFN Signaling. Corneal epithelial cells, stromal fibroblasts, and resident and infiltrated immune cells such as neutrophils, DCs, and macrophages express IFNAR. However, IFNLR is mainly expressed by corneal epithelial cells and infiltrating neutrophils. The activation of IFNAR and IFNLR results in the phosphorylation of STAT1/2, which then recruits IRF9 to form the ISGF3 complex. The ISGF3 complex translocates to the nucleus to bind to ISRE to induce the expression of ISGs. Macrophages and NK cells express IFNGR, which phosphorylates STAT1. STAT1 then forms a homodimer, also known as GAF. In the nucleus, GAF binds to GAS to induce the expression of ISGs. (Created with BioRender.com).

HSV-1 recognition through PRRs and downstream signaling initiates a robust production of type I and type III IFNs depending on cell type and site of virus replication [21,22,30,31]. These innate antiviral IFN responses are critical to control HSV-1 replication at infected sites, and they also contribute to the activation of adaptive immune responses and associated immunopathology in the cornea [21,22,23,30,31,153]. IFNs are divided into three families: type I, II, and III IFNs [154,155]. The type I IFN family consists of a single IFN-β, multiple IFN-α subtypes (13 in humans and 14 in mice), IFN-ε, IFN-κ, IFN-ω (humans), and IFN-ζ (mice) [33,156,157]. Type I IFNs bind and signal through the heterodimeric IFN-α/β receptor (IFNAR), consisting of IFNAR1 and IFNAR2 subunits (Figure 3) [33,34,156]. Type I IFNs bind with high affinity to IFNAR2, which promotes heterodimerization and the recruitment of low-affinity IFNAR1 subunits [33,34,156]. In humans, type III IFNs consist of 4 subtypes: IFN-λ1 (IL-29), IFN-λ2 (IL-28A), IFN-λ3 (IL-28B), and IFN-λ4 [34,158]. In mice, the type III IFN family is comprised of IFN-λ2 and IFN-λ3, whereas IFN-λ1 is a pseudogene, and the genomic region encoding the IFN-λ4 gene is absent [34,158,159,160]. Type III IFNs signal through the heterodimeric IFN-λ receptor (IFNLR), which consists of IFNLR1 (IL-28Rα) and IL10Rβ. IFN-λ binds with high affinity to IFNLR1, followed by recruitment and binding to the low-affinity IL10Rβ subunit [34,158,159]. 

IFN-λ is the frontline antiviral cytokine at mucosal epithelial surfaces and acts to elicit a robust antiviral response with limited inflammatory consequences [34,154,155,161,162]. Among type I and type III IFNs, IFN-λ acts as a first line of antiviral defense and is rapidly produced (as early as 4–6 h) by infected epithelial cells [30,34,163]. In contrast, the kinetics of IFN-α/β expression is slower and peaks after initial IFN-λ expression [34]. Another critical factor that affects the type I and type III IFN-mediated antiviral versus immuno-regulatory responses is the non-overlapping expression of their respective receptors. All nucleated cells express IFNAR [34]. In contrast, only mucosal epithelial cells and a subset of immune cells, such as neutrophils and DCs at barrier surfaces, express the IFNLR [158,164,165,166,167]. Cell surface TLRs such as TLR2 and TLR5 and peroxisome-associated MAVS signaling after viral infections promote IFN-λ production by myeloid and epithelial cells at mucosal barriers [161,168,169]. The restricted expression of IFNLR at mucosal barrier surfaces and early activation kinetics suggest a hierarchy of innate IFN responses after viral infections [158]. Signaling through both the IFNAR and IFNLR can promote the redundant expression of ISGs by infected and nearby uninfected cells, which results in diminished viral replication [170]. Thus, during the early stages of viral infection, localized and less potent IFN-λ responses are initiated in infected mucosal epithelial cells, which in turn induces a subset of ISGs compared to type I IFNs [171,172,173]. However, once the epithelial barrier is breached, the more powerful type I IFNs are initiated both locally and systemically, and this response acts predominantly to inhibit the subepithelial and systemic spread of the virus infection [33,34,156,161,162]. Another distinctive feature between IFNAR- and IFNLR-mediated signaling is the kinetics and magnitude of ISG induction. Type III IFNs promote more sustained expression of ISGs to limit local viral replication, whereas type I IFN-mediated ISG expression peaks early and declines rapidly due to possible negative regulation by inhibitory ISGs such as ISG15 and ubiquitin-specific protease (USP)17 [174,175,176,177,178]. In conclusion, the less potent type III IFN responses are predominantly induced at mucosal barrier surfaces during the early stages of virus infection. These IFN-λ-mediated antiviral responses are less inflammatory with slower and prolonged kinetics of ISG expression. Subsequently, more potent and more inflammatory type I IFN responses are induced locally and systemically with rapid kinetics to limit systemic virus spread. These distinct antiviral and less inflammatory features of type III IFN responses represent a promising therapeutic target during HSV-1-induced HSK to control viral replication and at the same time minimize the unwanted inflammatory implications that can culminate in vision loss and blindness [21]. 

### Type I and Type III IFN-Mediated Signaling

As discussed above, except for a few differences (site, kinetics, and magnitude), both type I and type III IFN-mediated signaling induce a comparable antiviral response characterized by the redundant expression of a similar set of ISGs, inflammatory cytokines, and chemokines [34,154,155,160,179]. Despite the structurally distinct IFNAR and IFNLR receptors and their different lFN ligands, the downstream signaling events and transcriptional antiviral responses mediated by IFN-α/β and IFN-λ show close similarity [34,154,155,160]. As mentioned earlier, IFNs first bind with high affinity to one receptor chain (IFN-λ to IFNLR1 and IFN-α/β to IFNAR2) followed by the recruitment of a low-affinity receptor chain (IFNAR1 and IL10Rβ) to form a signaling ternary complex (Figure 3) [34]. Subsequently, the ligand-engaged receptors activate the Janus kinase (JAK)-signal transducer and activator of transcription (STAT) signaling pathway [34]. JAKs are tyrosine kinases comprising JAK1, 2, and 3 [180]. IFN-α/β and IFN-λ use JAK1 and tyrosine kinase (Tyk) 2, and JAK1 for productive downstream signaling [34,156,181], whereas type II IFN (IFN-γ) binds to the type II IFN receptor (IFNGR) and activates JAK1 and JAK2 [34,155,158,181]. After engagement of the respective receptors, IFNs cause heterodimerization of the receptors leading to cross-activation of JAKs [34]. Activated JAKs phosphorylate specific tyrosine residues on the receptor polypeptides, which enables the docking of STAT proteins [34,155,181]. Once STATs are phosphorylated at particular tyrosyl residues by JAKs, STATs dissociate from the receptor, form heterodimers, and enter the nucleus [34,155,181]. IFN-α/β- and IFN-λ-mediated signaling phosphorylate and activate STAT1 and STAT2 to form a heterodimer, which subsequently interacts with IRF9 [34,181]. The activated STAT1–STAT2–IRF9 complex, also known as IFN-stimulated gene factor 3 (ISGF3), binds to the IFN-stimulated response element (ISRE), which is present in the promoter regions of many genes [181]. However, both type II IFNs and type I IFNs can activate STAT1 to form a STAT1–STAT1 homodimer, also known as a gamma IFN activation factor (GAF), which translocates into the nucleus and binds to gamma IFN activation sites (GASs) [181]. Type II IFNs are produced by NK cells and T cells but not directly by infected cells and, therefore, will not be further discussed in the review [179,182]. 

IFNs activate several genes to control viral replication and recruit and activate other immune cells through increased production of chemokines and cytokines [33,34,183,184,185]. Apart from STAT1 and 2, type I and III IFNs activate STAT3 as well as the phosphatidyl inositol 3 kinase (PI3K) pathway [186,187,188]. Type I and III IFNs also activate all three major mitogen-activated protein (MAP) kinases-p38, extracellular signal-regulated protein kinase (ERK1/2), and Jun N-terminal kinase (JNK) pathways [181,188]. Type I IFNs can also activate the mammalian target of rapamycin (mTOR) and its downstream factors, such as the ribosomal S6 kinase and eukaryotic translation initiation factor 4E-binding protein (eIF4E-BP), which control the translation of ISG gene products [189]. Unlike type I IFNs, type III IFNs can activate STAT4 in some cell types [190]. The induction of JAK inhibitory proteins, such as the suppressor of cytokine signaling (SOCS) terminates STAT activation [191]. Although IFNs are known to activate several pathways, the exact targets and their respective functions still need to be identified. Recent studies suggest that interaction among these signaling pathways may be required to mount an efficient innate immune response that, in turn, shapes the nature of the subsequent adaptive immune response [27,34]. Further studies are needed to understand the importance of the cross-talk between these signaling pathways with respect to a specific cell, organ, and disease type. The scheme of events and potential outcome is shown in Figure 3.

## 5. Anti-HSV-1 ISGs

HSV-1 recognition by PRRs drives robust IFN secretion, which in turn acts in an autocrine and paracrine manner to promote the expression of ISGs, PRRs, chemokines and cytokines [31,184]. Type I and III IFNs can induce more than 300 ISGs, promoting a robust antiviral state [183,184,192]. Despite numerous ISGs generated by IFNs, HSV-1 has evolved multiple evasion strategies to counteract or avoid many of their antiviral effects [40,41,42]. Among the hundreds of ISGs, a subset of ISGs play a critical role in controlling HSV-1 replication and infection. These include cholesterol-25-hydroxylase (CH25H), IFN-induced transmembrane protein 1 (IFITM1), myxovirus resistance protein (Mx)A, MxB, dsRNA-dependent protein kinase (PKR), 2’5’-oligoadenylate synthase (OAS)/RNase L, ISG15, virus inhibitory protein endoplasmic reticulum-associated interferon inducible (viperin), and tetherin [31,40,184]. The role of type I IFNs and anti-HSV-1 ISGs have been extensively reviewed elsewhere [31,40,41,183,184]. Here, we will briefly discuss the molecular mechanisms of action for some of these ISGs and their role in HSV-1 infection.

CH25H: CH25H is an enzyme that converts cholesterol into 25-hydroxycholesterol (25HC) [193]. The enzyme is cell membrane permeable and can inhibit sterol biosynthesis in both an autocrine and paracrine manner [193]. In addition, the sterol biosynthesis pathway is critical for isoprenoid generation and protein prenylation, a post-translation modification that affects viral proteins and the replication of many viruses [193,194,195]. Recent studies indicate that 25HC alters the host cell membrane which directly inhibits viral envelope fusion with the cell membrane, thereby inhibiting HSV-1 infection and replication [196,197]. 

IFITM1: Similar to 25HC, IFITM proteins affect viral fusion and entry. IFITM proteins are present in the endolysosomal compartment and affect the replication of viruses transiting through this route during its life cycle [183,184]. IFITM1 can localize to the plasma membrane, and as a recent study showed, IFITM1 can block HSV-1 entry via the plasma membrane and inhibit replication [198]. 

MxA and MxB: MxA and MxB (also called Mx1 and Mx2) are IFN-inducible GTPases that affect the early stages of viral genome replication by inhibiting viral capsid transport to the target cellular location [183,184]. Other suggested mechanisms include Mx1-oligomer ring formation and targeting of nucleocapsids for degradation [199,200,201]. During HSV-1 infection, plasmacytoid DCs (pDCs) can stimulate the production of MxA from nearby HSV-1-infected epithelial cells in the dermis [31,202]. Interestingly, HSV-1 promotes the expression of a spliced isoform of MxA, which is a 56-kDa protein and supports HSV-1 replication, instead of the MxA (76-kDa), which restricts HSV-1 replication [203]. Our recent study showed that corneal HSV-1 infection upregulates Mx1 expression during the early viral replication phase [21]. Moreover, MxB is an IFN-inducible anti-HSV-1 factor that inhibits HSV-1 replication in numerous human epithelial and neuronal cell lines [204,205]. 

PKR: During virus replication, dsRNA activates PKR [206]. Since viruses completely depend on host ribosomes for viral mRNA translation and protein synthesis, many ISGs, including PKR, target these steps [206]. For example, PKR phosphorylates eukaryotic translation initiation factor-2α (eIF-2α) and, following binding to viral dsRNA, inhibits viral protein synthesis [206]. PKR is critical for IFN-mediated resistance in mouse primary TG culture after HSV-1 infection [207]. Alternatively, HSV-1 infection suppresses the constitutive expression of PKR to escape antiviral immunity [208]. 

OAS/RNase L: OAS binds to dsRNA to promote the synthesis of 2′, 5′-oligoadenylate, which binds and activates latent RNase L [209,210,211,212]. Similar to PKR, OAS/RNase L plays an important role in inhibiting HSV-1 replication in murine primary TG neurons [207,213]. HSV-1 US11 protein inhibits OAS synthesis in IFN-stimulated primary human cells [214].

ISG15: ISG15 is a ubiquitin-like protein that plays an important role in protein post-translational modification through covalent attachment to the target proteins in a process called ISGylation [215]. ISGylation of IRF3 inhibits ubiquitin-mediated degradation and this promotes stability and transcription factor activity [216]. One past study showed that ISG15 is a critical anti-HSV-1 ISG, and ISG15 knockout mice cannot control HSV-1 infections [217]. Recently, we demonstrated that ISG15 expression is upregulated in HSV-1-infected corneas during the initial viral replication phase and that, in addition, IFN-λ significantly upregulates ISG15 expression in HSV-1-stimulated neutrophils to inhibit virus replication [21]. 

Viperin: Viperin is also called Cig5 or RSAD2, and this molecule inhibits numerous enveloped viruses through various antiviral mechanisms based on the viral replication cycle [183,184]. Viperin was first discovered as an IFN-γ-inducible protein after human cytomegalovirus (HCMV) gB stimulation [218]. Viperin inhibits the replication of numerous RNA and DNA viruses [218,219]. Recent studies indicate that HSV-1 proteins such as UL41 or virion host shutoff (vhs) can counteract viperin’s antiviral activity to promote HSV-1 replication [219,220,221]. HSV-1 vhs, an endoribonuclease, degrades viperin mRNA accumulation and inhibits viperin’s antiviral activity [219]. 

Tetherin: Tetherin is an ISG that acts during the late stages of virus replication and inhibits virus progeny particle budding from infected cells [222]. As the name indicates, this ISG tethers progeny viral particles to inhibit their release [222]. Recent studies suggest that tetherin restricts HSV-1 spread, and viral proteins such as gM and vhs antagonize tetherin activity [136,223,224]. 

Collectively, innate IFN responses post HSV-1 infection promote the induction of ISGs that can restrict HSV-1 replication. Alternatively, HSV-1 expresses numerous proteins that can directly counteract innate IFN and ISG responses. In the next section, we briefly review HSV-1 mediated immune evasion strategies to suppress innate IFN responses. However, many of these studies were performed using mutant HSV-1 viruses and cell lines, and the effector mechanisms are still unexplored during primary and recurrent corneal HSV-1 infection. The role of ISGs and their mechanism of action in suppressing HSV-1 replication is summarized in Table 1.

## 6. HSV-1 Evasion Strategies to Control Innate IFN Responses

The effective induction of antiviral immune responses mediated by type I IFNs and type III IFNs protect against HSV-1 infection in peripheral tissues, but ultimately HSV-1 enters the sensory nerve endings at the infection site and is transported retrogradely by physiological axonal transport mechanisms to the peripheral nerve ganglia [225,226]. Past studies on axonal transport mechanisms suggest that viral components such as capsids without envelopes and glycoproteins are transported separately and later assembled in the axon termini, rather than intact virion transport [227,228,229,230,231,232]. In the case of corneal and orofacial infection, HSV-1 undergoes latency in the TG [8,226]. It is still unclear why HSV-1 productively replicates in some neurons, causing neuronal and nearby satellite cell death, but establishes latency in other neurons with the expression of latency-associated transcript (LAT) but no viral proteins [7,225,233,234]. Similarly, it is still poorly understood why reactivation from latency occurs in only a subset of latently infected neurons [234].

**Figure 4:**
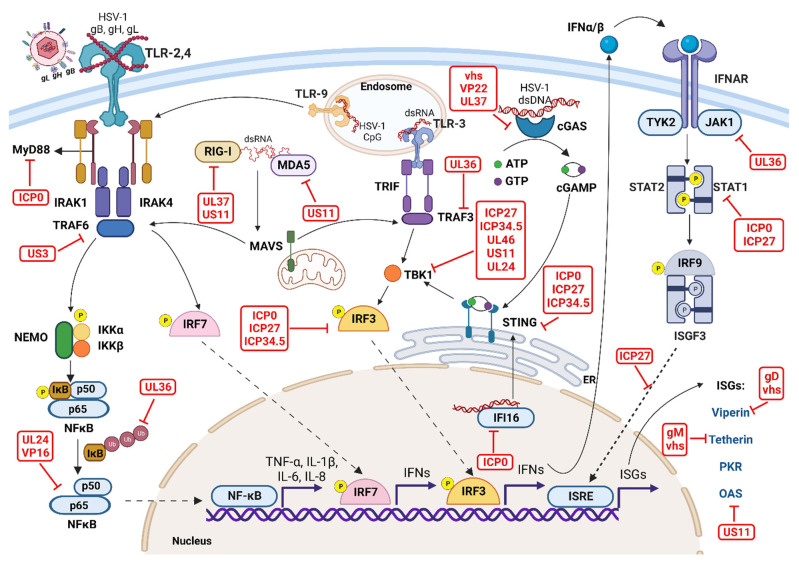
The role of HSV-1 proteins in the regulation of host antiviral immune responses. HSV-1 proteins target multiple steps after PRR activation to evade the host immune response. Further, HSV-1 can directly inhibit DNA sensors such as cGAS and IFI16 and RNA sensors such as RIG-I and MDA-5. HSV-1 directly or indirectly inhibits MyD88, TRAF6, TRAF3, STING, TBK1, and NF-κB to prevent cytokine and IFN production. HSV-1 also inhibits the IFN signaling pathway by blocking JAK1 and STAT1 phosphorylation, inhibiting ISGs. Further, HSV-1 proteins directly inhibit ISGs such as viperin, tetherin, and OAS to evade host antiviral immune responses. (Created with BioRender.com).

However, HSV-1 reactivation from latency frequently occurs, and this is more likely to be clinically evident in persons with a compromised immune system [6,8]. It is far from clear what causes reactivation and why its clinical consequences can be so variable. However, many have advocated that the HSV-1’s ability to evade one or more aspects of innate and adaptive immunity could be a relevant factor in the decision process [40,41,42,235]. Many investigators have identified several components of HSV-1 which can interfere in some way with the effectiveness of some aspects of immunity. This is commonly referred to as immune evasion. However, the evasion is never more than partial. For example, multiple viral proteins produced during the HSV-1 replication cycle interfere with several events. These include PRR-mediated recognition of HSV-1, PRR signaling, the production of type I and III IFNs, IFN-mediated signaling and production of ISGs, and antiviral effector functions of ISGs [42,236]. Furthermore, viral proteins such as ICP0, ICP27, and VP inhibit multiple PRR-mediated signaling pathways and type I IFN-mediated effector antiviral responses. Many reviews have extensively covered the topic of immune evasion strategies used by HSV-1 [31,40,41,42]. The following subsections will only briefly discuss how HSV-1 proteins interfere with the host’s innate antiviral responses during the HSV-1 life cycle (Figure 4, Table 2).

### 6.1. Evasion of PRR Signaling to Suppress Innate IFN Production

As discussed earlier, multiple PRRs (cell surface, endosomal, and cytosolic) are activated by different HSV-1-associated PAMPs to induce the robust production of IFNs and inflammatory cytokines (Figure 3). Alternatively, HSV-1 expresses several proteins that modulate multiple arms of PRR-mediated signaling events to suppress innate antiviral responses (Figure 4, Table 2).

Regulation of TLR-signaling-mediated anti-HSV-1 immunity: ICP0 is an IE protein that inhibits innate immunity via numerous mechanisms. For example, ICP0 is a viral E3 ligase that targets MyD88 and Mal for cellular proteasomal-mediated degradation and thus inhibits IFN production by infected cells [237,238,239,240]. ICP0 also promotes the cytoplasmic translocation of USP7 from the nucleus, where it binds and deubiquitinates TRAF6 and IKKγ to terminate TLR-mediated NF-κB and JNK activation [241]. During the early stages of HSV-1 replication, US3, a serine/threonine protein kinase, inhibits TRAF6 polyubiquitination and suppresses TLR2-mediated nuclear translocation of NF-κB and the production of inflammatory cytokines [242]. Using human monocytic cells, Peri et al. showed that US3 suppresses TLR3, type I IFN, and MxA mRNA expression [243]. In another study, Wang et al. showed that US3 phosphorylates IRF3 and inhibits IFN-β production to facilitate the evasion of host antiviral immunity [244]. Another HSV-1 tegument protein, VP16, interacts with the p65 subunit and blocks NF-κB-dependent genes, including the production of type I IFNs [245]. This study also showed that VP16 binds and selectively blocks IRF3 but not IRF7-mediated transactivation. VP16 did not affect IRF dimerization or nuclear translocation but did inhibit type I IFN production acting by interfering with IRF3 and its coactivator, cyclic adenosine monophosphate response element binding protein (CREB-BP) [245]. A recent study showed that UL24 binds to endogenous p65 and p50 subunits in HSV-1-infected cells and then inhibits NF-κB-mediated type I IFN production [246]. HSV-1 tegument protein UL36 has a novel deubiquitinase activity in its N terminus, called UL36USP. This enzymatic activity suppresses IFN-β production by deubiquitination of TRAF3 and destabilizes the polyubiquitin scaffold critical for downstream recruitment of TBK1, IRF3 dimerization, and activation [247]. HSV-1 UL42 interacts with IRF3 and inhibits its phosphorylation resulting in diminished IFN-β gene expression [248]. Accordingly, HSV-1 proteins have several mechanisms that permit the virus to evade immune control, and many of these are listed in Table 2.

Regulation of cGAS–STING-mediated anti-HSV-1 immunity: A recent study showed that vhs, through its RNase activity, targets cGAS mRNA for degradation [249]. Another study showed that UL56 binds with cGAS to inhibit its dsDNA-binding and enzymatic activity [250]. Similarly, VP22 inhibits the enzymatic activity of cGAS and suppresses type I IFN production [251]. VP22 also promotes the liquid condensation of viral dsDNA and creates phase separation of DNA and cGAS, inhibiting its activity [252]. Recently, it was shown that HSV-1 tegument protein UL37 has deamidation activity and promotes deamidation of human and mouse cGAS, which in turn affects the synthesis of cGAMP and suppresses IFN production [253]. RNase activity of vhs promotes the selective degradation of host cell mRNA molecules and thus decreases protein synthesis of the host’s antiviral effector molecules [249,254]. UL24 inhibits dsDNA-mediated cGAS–STING activation and IFN-β and IL-6 production during HSV-1 infection [246]. In addition, UL36USP suppresses cGAS–STING-mediated IFN-β promoter activation and blocks NF-κB activation through inhibition of IκBα degradation by deubiquitinating it [255]. Furthermore, UL36USP/VP1-2 also deubiquitinates STING and inhibits the activation of IRF3 and downstream induction of type I IFNs in murine and human microglia [256]. A recent study showed that the UL46 protein, through its N terminal domain, interacts with STING and, through its C terminal domain, interacts with TBK1. This suppresses STING activation and the production of IFNs during HSV-1 infection [257]. Another HSV-1 protein, ICP34.5 (encoded by γ_1_34.5 gene), associates with STING and disrupts the translocation of STING from the ER to the Golgi and inactivates STING-mediated anti-viral responses [258]. UL46 binds with TBK1 and impairs the interaction with IRF3 to downregulate type I IFN production [259]. In HSV-1 infected human macrophages, ICP27, through its RGG motif, interacts with TBK1 and suppresses STING-mediated type I IFN production [260]. Furthermore, Orzalli et al. showed that ICP0 relocalized in the nucleus targets IFI16 for degradation, inhibiting nuclear sensing of viral DNA and activation of IRF3 [261]. Similarly, another study showed that ICP0 promotes proteasome-mediated degradation of IFI16 and suppresses IFI16-mediated antiviral responses [262]. Although this study showed that ICP0 promotes IFI16 degradation, inhibition of IFI16-mediated responses was not completely dependent on ICP0. Moreover, another study showed that in addition to ICP0-mediated degradation, vhs-dependent targeting of IFI16 mRNA promotes complete loss of IFI16 in tumor-derived human cells [263]. 

Regulation of RLR-mediated anti-HSV-1 immunity: US11 is an RNA-binding tegument protein in HSV-1-infected cells that binds to endogenous RNA sensors, including RIG-I and MDA-5 [264]. US11, through the C-terminal RNA-binding domain, interacts with RIG-I and MDA-5 and interferes with the downstream interaction with MAVS and the production of IFN-β [264]. The deamidase activity of UL37 also targets RIG-I signaling to inhibit antiviral immune responses and prevent viral replication [265]. UL37-mediated deamidation in the helicase domain of RIG-I makes it unable to bind to viral dsRNA, inhibiting downstream activation of innate antiviral responses [265]. 

### 6.2. Evasion of IFN-Mediated Signaling

Initial studies suggested that HSV-1 blocks innate IFN responses and actively inhibits IFN-mediated downstream signaling at multiple sites [40,41,42,266]. During the early phase of HSV-1 replication, levels of IFNAR and JAK1 rapidly decrease, as do other STAT proteins, or are post-translationally modified, resulting in the suppression of ISGF3 formation [266]. This study identified HSV-1 vhs as partly responsible for this diminished IFN signaling [266]. UL36USP, in addition to deubiquitinating TRAF3 and STING, also inhibits type I IFN-mediated signaling [267]. UL36USP acts by binding directly to IFNAR2, and this blocks downstream recruitment of JAK1 and activation of STATs and the ISRE promoter [267]. ICP0 inhibits STAT-dependent host antiviral responses downstream of IFN signaling [268]. Johnson et al. showed that ICP27 inhibits STAT1 phosphorylation and nuclear translocation to suppress IFN-mediated expression of ISGs and consequent anti-HSV-1 immunity [269]. A further study from this group showed that ICP27 expression promotes the secretion of heat-stable type I IFN-antagonizing protein, which inhibits the STAT1 phosphorylation and nuclear accumulation [270]. The effects were noted at or upstream of JAK1 phosphorylation during type I IFN-mediated signaling [270].

**Table 2:** HSV-1 molecules interfere with host’s innate antiviral responses during the HSV-1 life cycle.

Host Responses	Host Target Molecule	HSV-1 Molecule	Mechanism of Action	References
TLR signaling	MyD88	ICP0	ICP0 degrades TLR adaptor proteins (MyD88 and Mal) to inhibit type I IFN production	[237,238,239,240]
	TLR2	US3	US3 reduces TRAF6 polyubiquitination to inhibit TLR2-mediated NF-kB activation	[242]
	TLR3		US3 suppresses TLR3-mediated type I IFN production	[243]
	p65	ICP0	ICP0 mediates USP7 translocation to cytoplasm, which induces deubiquitination of TRAF6 and IKKγ to terminate NF-κB activation	[241]
		VP16	VP16 interacts with p65 subunit to block the NF-kB activation and type I IFN production	[245]
		UL24	UL24 binds to p65 subunit to inhibit NF-kB mediated type I IFN production	[246]
	IRF3	US3	US3 phosphorylates IRF3 to inhibit IFN-β production	[244]
		VP16	VP16 interacts with IRF3 and its coactivator CREB-BP to inhibit type I IFN production	[245]
		UL42	UL42 inhibits IRF3 phosphorylation to reduce IFN-β gene expression	[248]
	TRAF3	UL36USP	UL36USP induces TRAF3 deubiquitination and destabilizes the polyubiquitin scaffold to suppress IFN-β production	[247]
dsDNA sensors	cGAS	UL24	Inhibits cGAS activation to inhibit IFN-β and IL-6 production	[246]
		vhs (UL41)	vhs targets cGAS mRNA for degradation	[249]
			vhs selectively degrades host antiviral effector molecule production	[249,254]
		UL56	Binds with cGAS to inhibit its dsDNA-binding and enzymatic activity	[250]
		VP22	Inhibits the binding of dsDNA to cGAS and suppresses type I IFN production	[251,252]
		UL37	UL37 promotes deamidation of cGAS to inhibit cGAMP and IFN production	[253]
		UL36USP	Inhibits cGAS–STING-mediated IFN-β promoter activation and blocks NF-kB activation	[255]
	STING	UL36USP	Deubiquitinates STING inhibiting IRF3 activation and type I IFN production	[256]
		UL46	Prevents STING activation to suppress IFN production	[257]
			Inhibits TBK1 dimerization to suppress IRF3 activation and type I IFN production	[259]
		ICP34.5	ICP34.5 blocks STING translocation from ER to Golgi to prevent its antiviral responses	[258]
		ICP27	ICP27 interacts with the STING-activated TBK1 to suppress type I IFN production	[260]
	IFI16	ICP0	Targets IFI16 for degradation to inhibit sensing viral DNA and IRF3 activation	[261,262]
RNA sensors	RIG-I	US11	US11 interacts with RIG-I to affect MAVS and IFN-β production	[264]
		UL37	UL37 deamidates RIG-I which affects its ability to sense dsRNA and inhibit antiviral immune responses	[265]
Type I IFN signaling	JAK1	vhs (UL41)	vhs reduces expression of IFNAR, JAK1 and STAT-2 to suppress ISGF3 formation	[266]
		UL36USP	UL36USP binds to IFNAR2 to block the recruitment of JAK1 and suppresses activation of STATs and the ISRE promoter	[267]
	STAT1	ICP0	ICP0 inhibits the STAT-dependent antiviral responses downstream of IFN signaling	[268]
		ICP27	ICP27 inhibits STAT1 phosphorylation and its nuclear translocation to suppress ISGs	[269]
ISGs	Viperin	vhs (UL41)	vhs reduces viperin mRNA accumulation to abrogate its antiviral effects and suppress viral replication	[219,220]
	Tetherin	vhs (UL41)	vhs depletes tetherin mRNA and protein in infected host cells to evade innate immune response	[224]
	OAS	US11	US11 dsRNA-binding domain blocks OAS synthesis and activation	[214]

### 6.3. Evasion of ISG Responses

As discussed earlier, type I and type III IFNs induce robust production of ISGs in HSV-1 infected and nearby uninfected cells to control viral replication. Conversely, HSV-1 has evolved strategies to neutralize ISG-mediated antiviral effector mechanisms. For example, vhs, by its endoribonuclease activity, targets viperin mRNA and thus reduces viperin levels, inhibiting HSV-1 replication [219,220]. vhs also targets tetherin mRNA and protein, thus promoting HSV-1 replication, dissemination, and infection of new cells [224]. The dsRNA-binding domain of US11 binds to OAS and inhibits its activity to promote HSV-1 replication in IFN-stimulated primary human cells [214].

Collectively, these studies suggest that during replication, the expression of HSV-1 proteins interferes with PRR signaling and downstream induction of IFN responses to evade antiviral immunity (Figure 4, Table 2). However, many of these molecular mechanisms were investigated using different in vitro assays, cell lines, and mutant HSV-1 viruses. The in vivo relevance of these immune evasion strategies in human HSK pathology and mouse primary and recurrent HSV-1 infection models is still poorly understood. 

## 7. Corneal HSV-1 Infection and Induction of Type I and III IFN Responses 

After corneal HSV-1 infection, both type I and III IFN responses are induced [8,21]. Type I IFNs (IFN-α and β) are mainly produced by DCs, infected corneal epithelial cells, and macrophages [22,97,271]. During HSK, type I IFNs induce a robust antiviral response in infected and nearby uninfected cells [22,23]. Type I IFNs also activate innate immune cells such as NK cells and macrophages to produce IFN-γ, which acts in an autocrine and paracrine manner to activate infected cells to promote HSV-1 clearance [154,272,273]. Compared to type I IFNs, corneal epithelial cells after HSV-1 infection predominantly produce IFN-λ [21,274]. These early type I and type III IFNs and downstream induction of ISG responses suppress viral spread in the cornea [21,22,97,275]. In the following subsections, we will discuss the current understanding of the role of IFN-α/β and IFN-λ after corneal HSV-1 infection and how these responses promote antiviral responses and regulate innate and adaptive immune responses during HSK progression. 

### 7.1. Type I IFNs after Corneal HSV-1 Infection 

As mentioned earlier, the type I IFN family consists of a multi-gene cytokine family. In this review, we will limit our discussion to the role of IFN-α and -β after corneal HSV-1 infection. Corneal HSV-1 infection stimulates IFN-α/β production in the infected epithelial cells and myeloid cells, such as DCs, neutrophils, and macrophages, to initiate antiviral responses in the cornea, which are necessary events for maintaining corneal integrity, normal lymphatic vessels, epithelial structure, and sensory excitation [9,22,276,277]. Corneal epithelial cells produce IFN-α through TLR-dependent and TLR-independent innate sensor mechanisms [136,141,278]. As discussed earlier, DNA sensors such as cGAS and IFI16 are critical for the activation of IRF3/7 to produce type I IFNs after HSV-1 infection [140,279,280]. Recently, Jamali et al. identified the role of cornea-resident pDCs for TLR-9-dependent IFN-α production after HSV-1 infection [97]. Local depletion of pDCs from the cornea increased both the viral burden and mortality after corneal HSV-1 infection [97]. The early induction of type I IFNs after corneal HSV-1 infection helps establish an antiviral state and promotes the recruitment of other immune cells into the cornea [22,97,281]. Previous studies using IFNAR knockout (CD118^-/-^) mice have examined the critical role of IFNs in controlling corneal HSV-1 infection [22,282]. 

In addition to the antiviral role played by the type I IFNs, these molecules are important for recruiting and activating other immune cells into the HSV-1-infected cornea [281]. Using CD118^-/-^ mice, Conrady et al. identified that IFN-α signaling is critical for producing the chemokine (C-C motif) ligand 2 (CCL2), which is responsible for recruiting inflammatory monocytes into the cornea [281]. Inflammatory monocytes produce nitric oxide (NO), which is critical for the containment of HSV-1 within the first 48 h of infection [281]. Following HSV-1 infection, type I IFNs induce the production of CXCL9 and CXCL10, which helps recruit NK cells and T cells into the cornea [128]. Furthermore, type I IFNs can enhance the maturation of DCs by upregulating MHC-II and co-stimulatory molecule expression, which is critical for Ag processing and presentation to T cells [28]. Additionally, type I IFNs affect the maturation, survival, and expansion of NK cells directly or indirectly through the modulation of other immune cells [272,283,284]. Without IFNAR signaling, inflammatory monocytes become deficient in IL-18 production, which leads to minimal NK-cell-mediated IFN-γ production and viral immunopathology [5,272]. However, IFNAR signaling in NK cells also can directly suppress NK-cell-mediated IFN-γ production [285]. Thus, type I IFNs play a critical role in regulating NK cell activation, preventing immunopathology associated with increased IFN-γ production [154]. Further studies are needed to understand the correlation between type I IFNs and IFN-γ production after HSV-1 infection.

### 7.2. Type III IFNs after Corneal HSV-1 Infection

Although the type III IFNs share less sequence homology with type I IFNs, type III IFNs exhibit several antiviral functions that are similar to type I IFNs [34,154,155]. Our previous study showed that HSV-1 infection induces IFN-λ expression in the cornea and that topical administration of recombinant IFN-λ suppresses HSV-1 replication in the cornea and HSK severity [21]. Furthermore, our study showed that IFN-λ regulates neutrophil antiviral function by increasing the production of ISG-15 and USP-18. Moreover, IFN-λ also suppresses the migration and activation of innate immune cells, such as neutrophils, NK cells, and macrophages, into the cornea by limiting the expression of inflammatory cytokines (IL-1β and IL-6) and chemokines (CXCL1 and CXCL10) [21]. Similarly, Jaggi et al. showed that systemic administration of PEGylated IL-28A during the early stage of HSV-1 infection suppresses the infiltration of neutrophils and Th1 cells in the cornea, alleviating HSK disease severity [275]. A recent study showed that IFN-λ and its receptor expression is increased in HSV-1-infected human corneal explants within 24 h, and blocking IFNLR leads to increased HSV-1 replication [274]. This study further showed that treatment with IFN-λ increases the ISG expression in human corneal explants [274]. When compared to type III IFNs, type I IFNs trigger a faster and stronger ISG induction along with an additional expression of pro-inflammatory cytokines and chemokines [34,286]. However, unrestrained expression of type I IFNs can cause severe corneal immunopathology, which limits the use of type I IFNs as a therapeutic approach for corneal HSV-1 infection [287]. In contrast, exogenous type III IFNs maintain the cornea’s antiviral state after HSV-1 infection, minimizing corneal immunopathology. Thus, the redundant antiviral role of IFN-λ with minimal inflammatory consequences and suppression of neutrophil-mediated inflammatory responses can be targeted therapeutically to suppress HSK progression.

## 8. Pathogenesis of Corneal HSV-1 Infection 

Once infected with HSV-1, the patient’s immune system is unable to completely eradicate the pathogen because of the latent infection in neurons [4,7,82,85,225]. Currently there is no cure for latent HSV-1 infection or successful vaccines that prevent new infections [7,288]. The HSV-1 latency is subject to temporary interruption which depends on still poorly understood factors such as immunosuppression, stress, ultraviolet (UV) exposure, and ocular surgical procedures. HSV-1 periodically reactivates and migrates anterograde in the sensory neurons to the innervated epithelial surfaces [225,226]. In humans, the primary corneal HSV-1 infection is rare, and most reported cases are recurrent infections because of the partial breakdown of latency [1,13,14,289]. Depending on the extent of HSV-1 replication, the severity of corneal cell layer damage, and the involvement of the host’s immune system, HSV-1-induced keratitis broadly can be classified as herpetic epithelial keratitis (HEK), HSK, and herpetic endothelitis [1]. 

HEK: HSV-1 predominantly replicates in the corneal epithelial cells but can also infect underlying stromal fibroblast cells. The HSV-1 replication and shedding in the cornea can be symptomatic or asymptomatic depending on the activation of innate antiviral responses and the immune control of virus replication [35,290]. The lytic HSV-1 replication in the corneal epithelium occurs within 12–24 h, and this leads to the development of punctate epithelial keratopathy characterized by granular spots [1,11,291]. The rapid HSV-1 replication, necroptic epithelial cell death, release of viral particles, and infection of nearby cells leads to the formation of raised dendriform lesions on the cornea [1,11,291,292]. The subsequent destruction of basement membrane leads to the formation of dendritic ulcers characterized by a branching linear pattern with large terminal bulbs [1,292]. This ulcer can further grow to form geographic ulcerative lesions on the corneal surface. 

**Figure 5:**
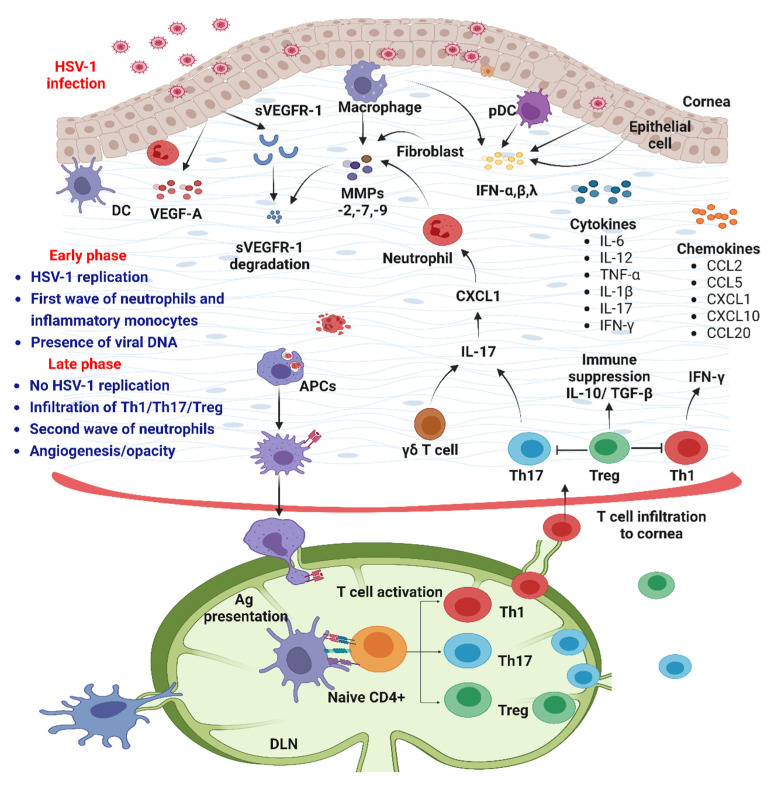
HSK immunopathology. In the pre-clinical or early phase, HSV-1 replicates in the corneal epithelium, which triggers the infiltration of innate immune cells, such as neutrophils and macrophages, into the cornea. Further, the production of chemokines, cytokines, and MMPs exacerbate inflammation and angiogenesis in the cornea. In the clinical phase of HSK, there is limited or no HSV-1 replication. However, APCs, such as macrophages and dendritic cells, induce T cell activation and recruitment to the cornea. Activated T cells, majorly comprising of Th1 and limited Th17 cells, infiltrate the cornea causing corneal immunopathology. (Created with BioRender.com).

HSK: HSK is an immunopathological disease involving the components of both the innate and adaptive immune system (Figure 5) [1,8,26]. Stromal keratitis can result from an immunological reaction to the replicating HSV-1 in the epithelium, as well as HSV-1 invasion and infection of stromal fibroblasts in the anterior stroma [293,294]. HSK can manifest as a necrotizing or non-necrotizing disease [1,8,291]. During the non-necrotizing keratitis, the inflammatory disease in the stroma can be focal or diffused with an intact epithelial layer [1,291]. Necrotizing keratitis involves the stromal infiltration of immune cells, hyperkeratinization of the epithelial layer, thickening and fibrosis in the stromal/epithelial layers, necrotic ulcers, edema, and abscesses often accompanied by secondary bacterial infections, causing thinning and corneal perforation in severe cases [1,291,295]. Most of our present understanding of HSK immunopathogenesis is based on studies performed using mouse and rabbit models of primary and recurrent HSV-1 infection and HSK [16,18,296,297]. 

Past studies using mouse models of primary and recurrent HSV-1 infection have defined HSK as an immunopathological condition mainly driven by viral replication in the corneal epithelium followed by uncontrolled activation of innate and adaptive immune responses (Figure 3) [16,18,26,298,299,300,301]. The mouse model of primary corneal HSV-1 infection mimics human HSK features which include corneal opacity caused by virus replication, immune cell infiltration, and the formation of new blood vessels in the otherwise normally avascular cornea [26,302]. Upon infection, the HSV-1 replicates in the corneal epithelium and the replicating virus is detectable in the cornea until day 7 pi, viral mRNA until day 7–8, and viral DNA is detected until day 20 pi [18]. The visible clinical features of HSK, such as opacity and angiogenesis in the mouse model, can be noted from day 7–8 pi [18,302]. Thus, in the mouse model there is a distinct early phase when the replicating virus is present in the cornea along with the infiltration of innate immune cells followed by the late phase characterized by opacity, angiogenesis, and infiltration of T cells and neutrophils in the cornea [16,17,18,302]. As discussed earlier, during the initial phase, HSV-1 is cleared from the cornea primarily through the induction of innate type I and III IFN-mediated antiviral responses [21,22]. However, the initial antiviral responses to control HSV-1 infection result in robust inflammatory responses that persist even after the virus is cleared off from the cornea [17,18]. 

During the early virus replication phase, neutrophils, macrophages, DC, γδ T cells, and NK cells infiltrate the cornea and play a critical role in HSV-1 clearance through multimodal mechanisms [8,18]. HSV-1 infected epithelial cells, stromal fibroblasts, and infiltrated immune cells produce several chemokines (CCL2, CCL5, CXCL10, CXCL1, and CCL20) and cytokines (IL-1, IL-6, TNF-α, IFN-γ, IL-12, and IL-17) that cause chronic inflammation [1,8,18]. HSK is considered as a T-cell-mediated immunopathology as mice depleted for T cells are less prone to HSV-1-induced HSK progression [298,299,301]. In both humans and mouse HSV-1 infected corneas, T cells infiltrate and orchestrate inflammatory and tissue-damaging responses [303,304,305]. Past studies from our group and others have shown that HSK lesions are mainly orchestrated by IFN-γ producing CD4^+^ T cells (Th1) and to a lesser extent by IL-17A^+^ CD4^+^ T cells (Th17) recognizing virus-derived peptides or unmasked self-Ags in the damaged cornea [298,299,300,306]. Moreover, our past studies have identified Foxp3^+^ CD4^+^ regulatory T cells(Treg)-mediated protection during HSK progression [307,308]. The role of these inflammatory cytokines and immune cells have been reviewed in detail elsewhere [8,18,297,303,309]. 

Another key feature of HSK pathogenesis is the neovascularization in the cornea [302]. The formation of new blood vessels from limbal blood vessels begins 24 h pi and gradually grow and peak at day 15 pi [18,302]. Although a normal cornea expresses vascular endothelial growth factor (VEGF)-A, its angiogenic activity is repressed through binding to the soluble form of VEGF receptor 1 (sVEGFR-1) [310]. Past studies have shown that HSV-1-induced corneal neovascularization is caused by increased VEGF-A production by infected and nearby uninfected corneal epithelial cells and infiltrating immune cells in the cornea [311,312,313,314]. Although VEGF-A/sVEGFR-1 balance in the normal cornea maintains avascularity [310], our past study showed that corneal HSV-1 infection dysregulates this physiological balance and promotes angiogenesis through increased VEGF-A and decreased sVEGFR-1 production [315]. Further we showed that HSV-1 infection in the cornea promotes the expression of sVEGFR-1-degrading metalloproteases (MMP)-2, MMP-7, and MMP-9 [315]. Our studies also revealed the critical role of IL-17A, an inflammatory cytokine, in causing VEGF-A/sVEGFR-1 imbalance in HSV-1-infected cornea [316]. IL-17A promotes neutrophil infiltration in the cornea through the increased expression of CXCL1 and the production of angiogenic molecules, such as VEGF-A, IL-6, and sVEGFR-1, degrading MMP production by the corneal stromal fibroblasts and neutrophils [316]. Thus, the induction of innate antiviral responses post corneal HSV-1 infection initiate a cascade of innate and adaptive immune cell responses that promote inflammation and angiogenesis causing vision impairment during HSK. 

## 9. HSK Therapeutic Strategies and Challenges 

The conventional therapeutic management of HSE and HSK relies on antivirals to suppress HSV-1 replication, corticosteroids to provide symptomatic relief of inflammation/pain, and surgical interventions in more severe cases to replace the infected/inflamed cornea [12,35,291,317,318,319,320,321,322,323]. 

Antiviral Therapies: Antiviral treatments using long-term acyclovir therapy have been used for over two decades as a first-line therapy to treat ocular HSV-1 infections in humans [291,319,320]. In the United States, currently three systemic (acyclovir, valacyclovir, and famciclovir) and two topical (trifluridine and ganciclovir gel) antivirals are available to treat recurrent ocular HSV-1 infection and HSK [35,319]. Acyclovir is a purine nucleoside analog, which binds and inhibits viral DNA polymerase to suppress HSV-1 replication [319]. Acyclovir selectively targets viral DNA polymerase and has a 200-fold higher affinity compared to its affinity for host polymerase [35,319]. The current antiviral treatment approaches for ocular HSV-1 infection are aimed to inhibit the recurrent infection [319]. Past clinical trials and studies in immunocompetent patients have shown that acyclovir (400 mg; twice a day) suppresses recurrent corneal HSV-1 infection compared to placebo treatment patients previously diagnosed with ocular HSV-1 infection [324]. Due to the lower bioavailability of acyclovir in topical formulations, acyclovir and several of its analogs are administered systemically (oral or intravenous injections) at higher doses [35,319]. However, acyclovir has limited corneal penetration, requires a very high oral dose, may promote HSV-1 latency, and its prolonged use can cause severe side effects [321,325,326]. An alternative antiviral, trifluridine, a pyrimidine nucleoside, is available in a topical formulation for corneal HSV-1 infection [318,327]. However, it requires frequent topical administration, usually 8–10 times a day, and causes ocular and nephrotoxicity if used for an extended period [318,327]. Furthermore, the use of high oral doses and long-term acyclovir treatment is a significant risk factor for developing drug resistance and the emergence of acyclovir-resistant HSV-1 strains [328,329,330,331,332]. A recent study showed a very high incidence rate of acyclovir-resistant HSV-1 strains (>25%) in HSK patients [331]. Although alternative antivirals such as cidofovir or foscarnet are available, their prolonged use causes severe side effects [333,334]. Notably, the primary concern is the emergence of multi-drug-resistant HSV-1 strains in HSK patients [330,331,332]. In conclusion, novel alternate antiviral treatment approaches to control HSV-1 replication and HSK progression are urgently required to treat patient suffering from vision-impairing HEK and HSK. 

Anti-inflammatory Treatments: Anti-inflammatory drugs such as corticosteroids are often paired with antivirals to suppress inflammation and the acute symptoms associated with HSK progression [12,35,317,319]. However, the long-term use of corticosteroids is associated with many side effects such as glaucoma, cataracts, delayed wound healing, corneal thinning, secondary opportunistic pathogen infections, and recurrent episodes of HSV-1 infection [35,317,319,321]. Alternatively, topical cyclosporin has also been used for steroid refractory HSK [335,336,337]. Another drawback that limits the use of these anti-inflammatory therapies is the global suppression of protective immune responses such as innate IFN responses. For example, past studies have shown that corticosteroids suppress NK and CD8^+^ T-cell-mediated protective antiviral responses. This is also a key risk factor for the reactivation of HSV-1 in latently infected TGs, where CD8^+^ T cells play a critical role in suppressing HSV-1 reactivation [26,338,339]. 

Corneal Transplantation: Corneal transplantation is the last course therapy for HSK patients suffering from necrotizing keratitis with the risk of corneal melting and perforation. Penetrating keratoplasty and anterior lamellar transplant are used to replace inflamed corneas using healthy donor corneas [340]. The surgical excision of corneas also severs the corneal sensory nerves and can cause HSV-1 reactivation and recurrent infection in the transplanted cornea. Thus, the risk of transplant rejection due to recurrence of HSV-1, inflammation, and angiogenesis limits the long-term benefits of these surgical interventions [293,341,342,343].

### 9.1. Alternate Experimental Approaches to Control HSV-1 Infection and HSK

Several studies from multiple groups using experimental animal models of corneal HSV-1 infection have proposed alternate treatment approaches targeting latency, HSV-1 replication, inflammation, Th1 cells, Treg cells, host cell immunometabolism, angiogenesis, as well as some other approaches. Here, we briefly summarize some of these recently explored experimental HSK treatment approaches.

Prevention of HSV-1 infection and replication: Jaishankar et al. showed the potential use of BX795, an inhibitor of TBK1, as a promising alternate therapy to control acyclovir-resistant strains of HSV-1 [344]. BX795 blocks HSV-1 protein synthesis by targeting Akt phosphorylation in infected cells [344]. Similarly, 4-phenylbutyrate (PBA), a chemical-chaperone-based potent alleviator of ER stress is also effective in controlling HSV-1 infection through inhibition of viral protein synthesis [345]. PBA mimics CREB3 silencing and inhibits the translocation of NF-κB to the nucleus, thereby suppressing HSV-1 replication [345]. Further synergizing PBA with antiviral drugs such as acyclovir minimizes the dosage of systemic antiviral required to control HSV-1 replication [345]. Past studies have shown that targeting HSV-1 entry using aptamers, cationic peptides, or humanized antibodies against gD can reduce HSV-1 entry and inhibit replication [319,346,347,348,349]. Cationic peptides bind to anionic heparan sulfate (HS) and inhibit HSV-1 binding to the host cell surface, thereby reducing the infection [350].

The quest for an effective HSV-1 vaccine remains elusive. The rationale for developing an effective HSV-1 vaccine is strong as more than 3.7 billion people under the age of 50 are estimated to be latently infected with HSV-1 [15]. Latent HSV-1 infection predisposes infected individuals to severe neurological conditions such as meningitis and encephalitis [351]. Moreover, HSV-1 infection of the CNS is considered as one of the factors that may enhance multiple sclerosis (MS) [352,353] and the late onset of Alzheimer’s disease (AD) [354,355,356,357]. These studies indicate a dire need to develop prophylactic and therapeutic vaccines and treatment strategies to control HSV-1 infection. However, the development of an anti-HSV-1 vaccine has been largely unsuccessful. A more comprehensive overview on an anti-HSV-1 vaccine has been discussed in recently published reviews [7,288].

Inflammation: As discussed earlier, HSK is a T-cell-mediated chronic immunopathological disease primarily orchestrated by Th1 cells and to a lesser extent by Th17 cells [299,300,301,306,358]. Alternatively, increasing the Treg responses over Th1 and Th17 cells has shown a therapeutic promise in controlling HSK progression [303,306,307]. Our past studies have shown that exogenous administration of endogenous lectins such as galectin-9 and galectin-1 selectively promote Treg responses and suppress Th1 and Th17 responses, and limit the development of HSK lesions in the mouse model [359,360]. In addition, targeting the aryl hydrocarbon receptor using TCDD (2, 3, 7, 8-tetrachlorodibenzo-p-dioxin), a synthetic ligand, diminishes HSK progression through selective induction of Treg responses over Th1/Th17 responses [308]. Another useful therapeutic approach is to exploit the differences in major metabolic pathways of each T cell phenotype, which in turn affects their effector functions. For example, inflammatory T cells often rely on glycolysis, whereas regulatory T cells rely on AMP-activated protein kinase (AMPK) and lipid oxidation for their energy. In this regard, HSK lesions were significantly suppressed when glucose utilization was limited using 2-deoxy-glucose (2-DG) during the late phase of HSK [361]. However, treatment with 2-DG during the early phase of infection increased the spread of HSV-1 to the brain, causing encephalitis [362]. Therefore, the regulation of glucose metabolism can be beneficial or detrimental depending on the stage of HSV-1 pathogenesis. Besides glucose metabolism, inflammatory and regulatory T cell subsets show differences in amino acid metabolism, especially glutamine. Inhibition of glutamine metabolism using 6-Diazo-5-oxo-l-norleucine (DON) during the clinical phase of HSK also reduced lesion severity [363]. DON treatment significantly reduced Th1/Th17 responses without affecting the regulatory T cell population. Further studies are needed to understand the effects of metabolic regulators, especially when used for long-term treatment. Additionally, neutralizing inflammatory cytokines such as IL-17A and IL-6 using monoclonal antibodies in mouse models of HSK has shown promising results in limiting HSK progression [306,364]. 

Other alternate approaches have proposed the use of endogenously produced lipid mediators such as resolvin E1 and neuroprotectin D1 to control inflammation and limit HSK progression [365,366]. Another interesting approach to suppress corneal inflammation is to alter the phenotype of macrophages that infiltrate the cornea during HSV-1 infection. The polarization of macrophages into M1 (classically activated, inflammatory) and M2 (alternatively activated, anti-inflammatory) determines the severity of inflammation [367,368]. M1 macrophages exacerbate inflammation, whereas M2 macrophages are often associated with the blockade of inflammatory responses and promote tissue repair [367,368]. Lee et al. showed that the addition of colony-stimulating factor 1 (CSF-1) DNA enhanced the development of M2 macrophages, which produced high levels of IL-10, TGF-β, and arginase-1, and helped in controlling HSV-1 mediated corneal inflammation [271]. However, M2 macrophages promote primary HSV-1 replication and latency [369]. On the other hand, M1 macrophages were found to play a critical role in inhibiting ocular HSV-1 virus replication [370]. Therefore, a homeostatic balance between M1, which promotes viral clearance during the early phase of HSV-1 infection, and M2, which suppresses inflammation during HSK, is critical. Collectively, these studies indicate that selective inhibition of inflammatory responses is a promising approach to suppress HSK progression and vision impairment. However, the clinical benefits of such alternate approaches in human HSK patients remains to be explored.

Angiogenesis: As discussed earlier, the imbalance in VEGF-A/sVEGFR-1 leads to blood vessel development in normal avascular cornea and promotes HSK progression [311,312]. These newly formed blood vessels are often leaky and contribute to the increased infiltration of T cells (Th1/Th17) and neutrophils during the late phase when the replicating virus is largely absent in the cornea [302,306,315,316]. Our past studies have shown that the neutralization of VEGF-A using antibodies or exogenous sVEGFR1 administration suppresses HSV-1-induced corneal neovascularization and HSK progression [306,315]. HSV-1 infection of the cornea also promotes the expression of MMPs, which degrade extracellular matrix and promote corneal neovascularization [315,371]. Accordingly, experimental approaches to suppress MMP activity have shown beneficial effects in limiting HSV-1-induced corneal neovascularization and HSK [315,371]. Similarly, other experimental approaches using a small molecule inhibitor of Src kinase downstream of VEGF-A/VEGFR2 signaling or recombinant Slit Guidance Ligand 2 (Slit2, a ligand for endothelial cell roundabout 4 receptor) showed diminished corneal angiogenesis and HSK lesion severity [372,373]. In addition, studies in the mouse model have shown that neutralization of IL-6 or IL-17A using antibodies suppresses angiogenesis and HSK lesion severity [313,316]. Thus, these studies suggest that blocking angiogenesis could represent a potential therapeutic approach to limit HSK progression. 

In summary, the pathogenesis of HSK is multifaceted, involving a complex interplay of HSV-1 replication, inflammation, and angiogenesis. The current monotherapy approaches either targeting virus replication or inflammation are partially effective, and long-term use can lead to serious side effects. Thus, the development of alternative mono or combination therapies to target both viral replication and inflammation may be more effective. In this regard, a better understanding of antiviral, protective, and pathogenic immune responses could lead to the identification of novel therapeutic targets for the development of better therapies to treat HSK. One such approach we favor is topical IFN-λ therapy which promotes robust antiviral responses with minimal activation of inflammatory responses. This preference is further developed in the next section.

### 9.2. Is IFN-λ-Based Therapy a Better Approach to Suppress Both HSV-1 Replication and Inflammation?

Our recent study demonstrated that topical IFN-λ treatment during ocular HSV-1 infection represents a better approach to control viral replication and inflammation. Specifically, this approach will be highly effective against HEK, where the replicating virus is still present. As discussed earlier, treatment with type I IFNs induces a strong inflammatory response that could exacerbate corneal immunopathology and limit its use as a therapeutic [287]. Alternatively, type III IFNs can control corneal HSV-1 replication without incurring a strong inflammatory response. Our study showed that early treatment with IFN-λ significantly suppressed HSV-1 replication in the cornea [21]. Additionally, IFN-λ upregulates the expression of USP-18, which in turn interacts with STAT2 to inhibit type I IFN signaling [21,374]. IFN-λ treatment in HSV-1 infected mice corneas upregulates antiviral ISG (IFIT2, IFIT3, and ISG15) expression levels as evidenced by the lack of responses in IFNLR1^-/-^ mice [274]. Additionally, IFN-λ can limit neutrophil-mediated inflammation by diminishing the recruitment of neutrophils into the cornea [21]. Although the role of IFN-λ during the early phase of corneal HSV-1 replication is well defined, the protective anti-inflammatory role during the late phase of HSK remains to be evaluated. 

In addition to inducing a robust antiviral state, IFN-λ also limits the inflammatory reaction through modulation of neutrophil biology. A recent study using a mouse model of rheumatoid arthritis showed that IFN-λ suppressed the numbers of Th17- and IL-17A-producing γδ T cells in the inflamed joint [375]. Furthermore, this study showed that IFN-λ treatment inhibits the recruitment of IL-1β-producing neutrophils and consequent inflammatory responses [375]. Similarly another study showed that IFN-λ protects mice against enteric viruses by inhibiting neutrophil-mediated ROS generation and degranulation [169]. Other groups have shown the protective role of IFN-λ against mucosal viral pathogens such as the norovirus and influenza A virus [162,171,376,377,378]. Similarly, we showed that exogenous topical treatment with IFN-λ suppresses inflammatory cytokines such as IL-6 and IL-1β production in HSV-1-infected corneas [21]. Our data showed that IFN-λ limits neutrophil infiltration in HSV-1-infected corneas through suppression of CXCL1 production [21]. However, the T cell specific activity of IFN-λ after corneal HSV-1 infection needs to be studied further. Although our data suggest that topical IFN-λ during the HSV-1 replication phase ameliorates HSK pathology, the effect is most likely through the inhibition of HSV-1 replication resulting in reduced infiltration of neutrophils and suppression of inflammation. Further studies are necessary to delineate if the mechanism involves HSV-1 evasion of IFN-λ-mediated antiviral responses at the ocular surface, as preliminary evidence supports. Moreover, the therapeutic benefits of IFN-λ during the late phase of HSK, when the replicating virus is absent in the cornea, remains to be explored. Nevertheless, our studies indicate that preventive topical IFN-λ therapy can be beneficial in patients suffering from recurrent HSK caused by multi-drug-resistant HSV-1 strains. Finally, topical IFN-λ therapy can serve as an adjunct therapy in combination with corticosteroids to simultaneously promote antiviral and anti-inflammatory responses. 

## 10. Concluding Remarks 

HSV-1 is a highly successful human pathogen that can cause or predispose immunocompromised infected people to some serious illnesses such as HSE, HSK, and AD. Despite tremendous advances in our understanding of the HSV-1 life cycle (entry, replication, innate IFN responses, immune evasion strategies, latency, and viral pathogenesis), recurrent infections in susceptible populations represent a major clinical problem. HSK is a chronic inflammatory disease involving innate and adaptive immune responses. Although the induction of robust innate IFN responses is critical to limit HSV-1 replication in the corneal epithelium, these IFN-mediated responses also activate the adaptive arms of immunity, promoting inflammation and vision loss. HSK pathogenesis is a multi-layered syndrome involving HSV-1 replication and the activation of protective innate antiviral responses, neutrophil and T-cell-mediated inflammatory responses, and angiogenesis. Unfortunately, current clinical therapies using antivirals and corticosteroids target only one arm of pathogenesis, are only partially effective, and have serious side effects. Recent advances in the immunometabolism field and the use of metabolic modulators to regulate inflammatory and antiviral responses represent a promising approach to limit inflammation and promote protective antiviral responses. However, it is still unknown whether HSV-1, through expression by its viral proteins, regulates immune cell metabolism to evade antiviral immunity. Thus, detailed studies are necessary to understand the direct and indirect role of viral proteins in the evasion of the host’s antiviral immunity. Moreover, there is an urgent need to identify clinical (diagnostic and translational) biomarkers and highly sensitive rapid screening assays/tests that can differentially diagnose ocular infections with different pathogens. Since the development of prophylactic and therapeutic vaccines against HSV-1 remains a major challenge, the successful control of HSK depends on finding novel mono or combination therapies that can inhibit viral replication, inflammation, and angiogenesis without limiting antiviral immune responses. The future studies on the development of novel antivirals or the repurposing of FDA-approved drugs that can limit HSV-1 replication, promote protective IFN responses or suppress inflammation offer a new hope. Similarly, topical IFN-λ therapy alone or in combination with anti-inflammatory drugs could have additive or synergistic beneficial effects to limit HSE and HSK progression. In conclusion, recent advances in our understanding of HSV-1-mediated immune evasion strategies and the host’s antiviral and anti-inflammatory responses could lead to the development of novel, safe, and effective HSK therapies. 

## Figures and Tables

**Table 1:** Mechanisms of action of ISGs and their roles in the HSV-1 life cycle.

ISGs	Mechanism of Action	Roles in HSV-1 Life Cycle	References
CH25H	Blocks viral fusion with the host cell membrane by altering cell membrane; inhibits viral proteins prenylation by converting cholesterol into 25-hydroxycholesterol (25HC)	Inhibits HSV-1 envelope fusion with the host cell membrane	[193,194,195,196]
IFITM1	IFITM1 proteins block viral fusion with the host cell membrane and also trap viral proteins in the endolysosomal compartment to inhibit viral replication	Blocks HSV-1 entry into host cell and inhibits its replication	[183,184,198]
MxA (Mx1) and MxB (Mx2)	Affects early viral replication by inhibiting capsid transport within the cells	MxA and MxB inhibits viral replication	[21,183,184,199,200,201,203,204,205]
	Forms MxA-oligomer ring for nucleocapsid degradation		
PKR	Binds to dsRNA and phosphorylates eukaryotic translation initiation factor-2 α (eIF-2α) to inhibit viral protein synthesis	Inhibits HSV-1 replication	[206,207,208]
OAS/RNase L	Binds to viral dsRNA to promote the synthesis of 2’,5’-oligoadenylate and activate latent RNase L to inhibit viral replication	Inhibits HSV-1 replication in neurons	[207,209,210,211,212,213]
ISG15	ISGylation of IRF3 prevents ubiquitin-mediated degradation, promotes stability and transcriptional activation	Inhibits viral replication	[21,215,216,217]
Viperin	Interacts with HSV-1 gD to promote IRF7 mediated IFN-β signaling	Inhibits HSV-1 replication	[183,184,219,220,221]
Tetherin	Restrains the release of virus progeny particles budding from infected host cells during the late stage of viral replication	Inhibits HSV-1 spread	[136,222,223,224]

## Data Availability

Not applicable.
